# Choline Salicylate Analysis: Chemical Stability and Degradation Product Identification

**DOI:** 10.3390/molecules25010051

**Published:** 2019-12-22

**Authors:** Katarzyna B. Wróblewska, Szymon Plewa, Paweł Dereziński, Izabela Muszalska-Kolos

**Affiliations:** 1 Department of Pharmaceutical Technology, Poznan University of Medical Sciences, Grunwaldzka 6, 60-780 Poznan, Poland; kzajac@ump.edu.pl; 2 Department of Inorganic and Analytical Chemistry, Poznan University of Medical Sciences, 6 Grunwaldzka Str., 60-780 Poznan, Poland; splewa@ump.edu.pl (S.P.); pderezin@ump.edu.pl (P.D.); 3 Department of Pharmaceutical Chemistry, Poznan University of Medical Sciences, 6 Grunwaldzka Str., 60-780 Poznan, Poland

**Keywords:** choline salicylate, stress test, ionic liquid, HPLC, UV, HPLC-MS/MS

## Abstract

Choline salicylate (CS) as a derivative of acetylsalicylic acid is commonly used in different drug forms. In medicine, it is applied topically to inflammation of the oral cavity mucosa and in laryngology. However, this substance in the form of an ionic liquid has not been investigated enough. There are no literature studies on stability tests constituting a stage of pre-formulation research. HPLC (Nucleosil C18, 4.6 × 150 mm, 5 μm; methanol-water-acetic acid 60:40:1, 230 nm or 270 nm) and UV (276 nm) methods for the determination of CS in 2% (g/mL) aqueous solutions were developed. Under stress conditions, CS susceptibility to hydrolytic degradation in aqueous medium, hydrochloric acid, sodium hydroxide, and hydrogen peroxide, and the effect of light on the stability of CS solutions were studied with HPLC analysis. The degradation degree of CS and the purity of the solutions were also tested. Choline salicylate has been qualified as practically stable in neutral and acid media, stable in an alkaline medium, very stable in an oxidizing environment, and photolabile in solution. The HPLC-MS/MS method was used to identify 2,3- and 2,5-dihydroxybenzoic acids as degradation products of CS under the tested conditions.

## 1. Introduction

Choline salicylate (CS) belongs to the group of non-steroidal anti-inflammatory drugs (NSAID) derived from salicylic acid. By inhibiting prostaglandin synthesis, it shows anti-inflammatory, analgesic, and antipyretic effects on the hypothalamic heat control center. The analgesic activity, associated with hindering the transmission of pain impulses, is the effect of the peripheral and central action. Used in higher doses, CS acts as an anticoagulant, which is antagonistic to vitamin K [1]. It is administered orally to reduce pain, swelling, and joint stiffness in rheumatic diseases in children and adults. The standard dose equal to 435 mg of CS corresponds to approximately 325 mg of acetylsalicylic acid. For the treatment of pain and fever, CS is used orally at a dose of 435–870 mg to be administered every few hours. In adult rheumatic diseases, the dose is 4.8–7.2 g per day, in divided doses (ARTHROPAN, Purdue Frederick, United States) [2]. CS is used topically in the form of 20% drops in laryngology for treating inflammation of the middle ear and for softening and removing wax (OTINUM, PharmaSwiss/Valean Poland, SIA Meda Pharma, Lithuania; OTOTALGIN Farmina, Poland; AUDAX 20% Mundipharma, Cyprus) [3,4,5].

However, CS is most frequently used in the form of 8.7% gel combined with cetalkonium chloride or benzalkonium chloride (SACHOL, PharmaSwiss/Valean Poland; PANSORAL, Pierre Fabre, Lebanon, Pierre Fabre Médicament, France; Rovafarm, Argentina; DENCOL, Berko, Turkey) and in the form of lozenges for the treatment of inflammation, pain, and swelling of the oral cavity and throat mucosa (CHOLINEX GlaxoSmithKline, Poland; FARINGODOL FORTE, GlaxoSmithKline, Lithuania, Omega Pharma, Estonia) [6]. In addition, CS is used topically for muscle pain, muscle injuries, and rheumatic pain [2,7].

Acetylsalicylic acid and its derivatives are not available as commercial ophthalmic preparations, mainly due to their insufficient stability in solutions [8]. However, scientists have reported that acetylsalicylic acid in the form of topical eye preparations is effective in the treatment of allergic inflammation, the reduction of post-operative inflammation, as well as the prevention and treatment of cystoids macular edema inflammation [9,10]. It has also been shown that acetylsalicylic acid (by blocking cyclooxygenase) has an antiproliferative effect in intracellular fibrogenesis, and it can therefore be used in the treatment of proliferative vitreoretinopathy and cataracts [7,11,12]. This fact may result in the attempt to use CS as a carrier of salicylic acid in preparations for ophthalmic therapy. Earlier studies have shown that CS can be used as an active substance in ophthalmic preparations. The eye irritation potential was determined using cytotoxicity test methods for the rabbit corneal cell line (SIRC) after 5 min of exposure. Cell viability was determined using two cytotoxicity assays: MTT and neutral red uptake. All eye drops tested were classified as non-irritants (cell viability > 70%). It was therefore concluded that CS will not irritate the eye after application to the conjunctival sac [13].

The side effects observed after the application of CS are significantly weaker and occur less frequently than with acetylsalicylic acid. Topical preparations containing CS may induce Reye’s syndrome in children under the age of 12, but only if such preparations are co-administered with other oral NSAID or in children with an accompanying viral infection [14]. There have been no cases of Reye’s syndrome in children if the gel is applied in small quantities only on the spot of inflammation affecting the oral mucous membrane. However, topical preparations are contraindicated in children under the age of 3 as well as pregnant and/or breast-feeding women [15]. Choline salicylate should be used with caution in patients with chronic renal failure, stomach ulcers, and severe anemia and in patients who are intolerant to salicylates. It is also contraindicated in patients with coagulation disorders and those who take anticoagulants [16].

Considering the chemical nature, CS is a salt featuring the properties of ionic liquid [17]. It is a highly hygroscopic, non-toxic, and biodegradable substance with a melting point of 49.2–50.0 °C. It dissolves very well in water and creates stable solutions in it. CS aqueous solutions with a 10% concentration have a pH of approximately 6.5 [18]. Choline salicylate dissolves well in ethanol, acetone, and other hydrophilic solvents, but it is practically insoluble in ether and other organic solvents [19]. Hence, it is convenient to develop a formulation as an alternative to salicylates and other NSAIDs. CS is obtained by reaction in the ethanol environment of acid choline salts, e.g., choline chloride and bromide, with alkaline salts of salicylic acid, e.g., sodium, potassium, and magnesium salicylate [19].

As a derivative of acetylsalicylic acid, CS is used for treatment in various drug forms: solid (e.g., tablets and lozenges), liquid (e.g., ear drops and nose drops), and semi-solid, such as gels. However, it is apparent from a review of the literature that the properties of this substance as an ionic-liquid salt are poorly understood. That is, comprehensive stability tests of CS as an active pharmaceutical ingredient (API) are lacking, including in particular the so-called “stress tests,” which, in accordance with the guidelines of the International Council for Harmonization (ICH, document Q1A R2), aim to assess the susceptibility of both the active substance and the pharmaceutical form to degradation factors. Such testing makes it possible to determine the appropriate storage conditions for APIs and avoid the conditions that jeopardize the stability of APIs in the prepared drug form, both during the manufacturing process and in the drug formulation [20]. Kumari et al. performed a stability analysis of choline magnesium trisalicylate (Trilisate) using a stress test without an analysis of the mechanism of the degradation or the effect of magnesium ions on the salicylate stability [21]. CS and Trilisate differ in physicochemical properties. Therefore, the objective of this study was to determine CS susceptibility to environmental factors (oxidation, photodegradation, and hydrolysis at an elevated temperature in acidic, neutral, and alkaline environments) and to identify the observed degradation products with the HPLC-MS/MS method, while developing and validating the HPLC and UV methods used to determine CS in solutions.

## 2. Results and Discussion

### 2.1. Method Validation

**Figure 1:**
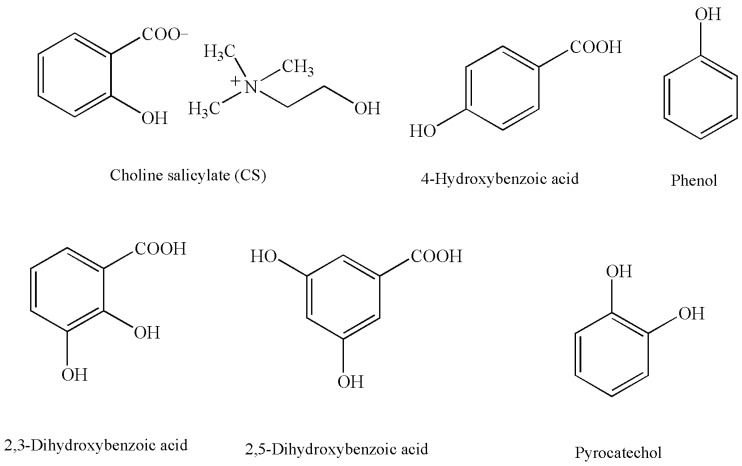
Structural formulas of choline salicylate (CS) and its possible impurities and degradation products.

As an ionic liquid, CS (Figure 1) has become an interesting source of salicylic acid, i.e., a pharmacologically active compound with limited physical-chemical properties to be used in drug formulation technology. The compound is very well soluble in an aqueous environment, which makes it necessary to develop a drug formulation in the form of a solution. For the evaluation of the possibility of such a drug form, the factors affecting CS stability in the aquatic environment must be determined in order to avoid possible degradation factors during the storage of the raw material or a specific drug form. From the view point of decomposition degree evaluation, it is necessary to apply a method for assessing raw material or preparation (solution) purity or to develop a methodology of CS determination in aqueous solutions at a 2% concentration of the active substance. For purity testing with a selective approach, the HPLC-RP method with UV detection (270 nm) was developed. For CS determination, the HPLC-RP method with UV detection (230 nm) was developed and compared with the UV spectrophotometry method (276 nm). The UV method is more useful in conditions of optimization with regard to the composition of the preparation being developed. Hence, both methods were validated.

**Figure 2:**
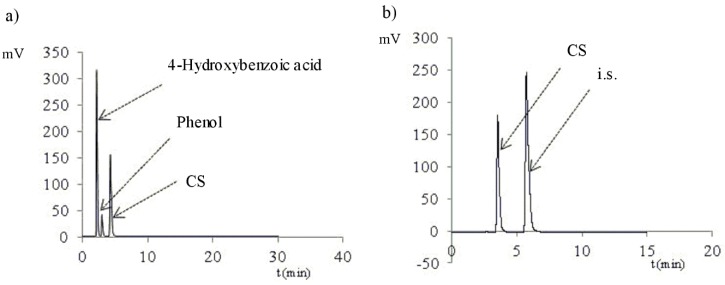
Exemplary HPLC chromatograms: (**a**) Mixtures of 4-hydroxybenzoic acid (50 µg/mL), phenol (50 µg/mL), and CS, (200 µg/mL)) and of (**b**) CS (80 µg/mL) and propyl 4-hydroxybenzoate (i.s., 100 µg/mL) are plotted against a mobile phase flow of 1.0 mL and detection at 270 nm (**a**) or 230 nm (**b**).

As far as the evaluation of selectivity of the HPLC method is concerned, the peaks of 4-hydroxybenzoic acid, phenol, and CS were separated under the chromatographic conditions described in Section 3.1.1. The peaks of the recorded compounds were observed with respect to the mobile phase flow (0.5 or 1.0 mL/min) in the following sequence: 4-hydroxybenzoic acid (t_r_ = 4.51 and 2.14 min), phenol (t_r_ = 6.24 and 2.92 min), CS (t_r_ = 8.85 and 4.08 min), and propyl 4-hydroxybenzoate (internal standard, t_r_ = 16.70 and 7.22 min) (Table 1, Figure 2). Phenol comprised 0.047% of the raw material tested. The potential contamination of CS requires no reporting, identification, or determination in accordance with the ICH Q3B Guidelines, Annex 1 (“Thresholds for degradation prodrugs in new drug prodrugs reporting thresholds”). Such contamination for the 2% CS solution will be <0.001%. Therefore, the raw material tested may be considered as conforming to the requirements of the ICH. Pyrocatechol, phenol, and 2,3- and 2,5-dihydroxybenzoic acid are described as oxidation and photodegradation products of salicylic acid [22,23]. Therefore, the selectivity of the purity analysis of CS solutions by HPLC-UV [Nucleosil C18 column (4.6 × 150 mm, 5 μm, mobile phase: methanol–water–acetic acid (60:40:1, *v/v/v*)), mobile phase flow 0.5 mL/min, UV detection at 270 nm] was determined. Under these conditions, the retention times of both the peaks of the potential impurities and the degradation product differ from the retention time of CS (Table 1). The presence of degradation products does not affect the result of the analysis of the CS content of the solution by HPLC-UV. The analysis of the qualitative composition of the CS mixture and degradation products requires the HPLC-MS/MS method (Table 2).

**Figure 3:**
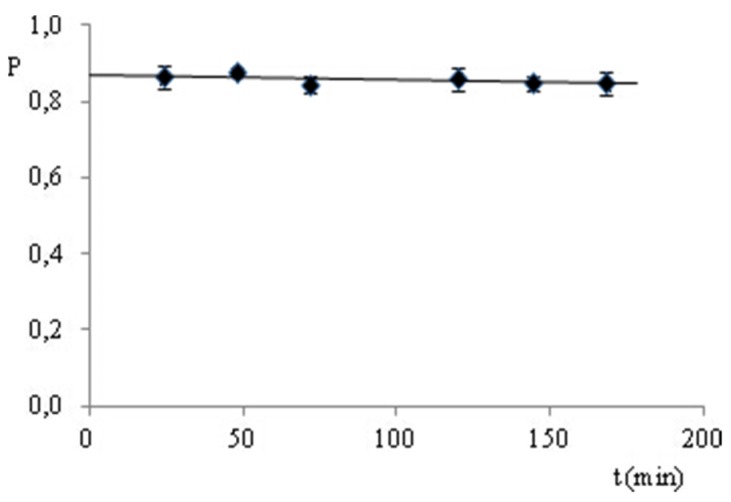
Plot of P as a function of time for a solution of CS (80 µg/mL) stored at room temperature under uncontrolled conditions protected from light.

For the determination of CS (2%) in solutions, an HPLC method with an internal standard (propyl 4-hydroxybenzoate) at a constant concentration of 100 µg/mL and with UV spectrophotometry (276 nm) was developed. The HPLC analysis was performed with a mobile phase flow of 1.0 mL/min and a UV detection of 230 nm. Standard curves were obtained with CS concentrations of 3.94–119.10 µg/mL (HPLC) and 2.52–80.56 µg/mL (UV), and are described by the equation y = *a*x (Table 3). With the use of the *t*-Student test, the lack of statistical significance of the coefficient *b* of the P or A_276_ relationships analyzed was proven to be a function of CS concentration (Table 3, *t_b_* < *t*_0.05_(7)). The calculated values of LOD, LOQ, and the *a* coefficients show that the UV method is more sensitive and that it is suitable for CS to be determined at a lower limit concentration of ≥1.30 µg/mL (Table 3). Both methods were validated by evaluating the linearity of the determinations within a concentration range of 50–150% of the declared value (2%), i.e., 1.00, 1.51, 2.01, 2.50, and 3.01%. The relationship of P or A_276_ as a function of CS concentration is described by the equation y = *a*x (Table 3). With the use of the *t*-Student test, the lack of statistical significance of the coefficients *b* of the relationships analyzed was proven (Table 3, *t_b_* < *t*_0.05_(7)). The values of the slope coefficients (*a*) confirm that the UV method is more sensitive (Table 3). The accuracy and precision of the HPLC and UV methods were evaluated on the basis of CS determinations in solutions with a concentration of 1.00, 2.01, and 3.01%. The CS content was calculated using the parameters of the standard curve (Table 4); the errors of the determinations were expressed as a confidence interval (± Δ). The accuracy was expressed as a percentage of the declared value. The calculated coefficients of variation (RSD, %) indicate that both methods comply with the criteria for precision requirements (RSD ≤ 1.86%), but the UV method is more precise (RSD ≤ 1.26%) (Table 4). The comparative analysis of both methods for evaluating accuracy (99.6–101.0%) can be summarized similarly. Both methods meet the validation criteria. The HPLC method was used to observe the stability of the analyte–CS solution (80 µg/mL) with an internal standard (100 µg/mL) in the mobile phase, stored at room temperature, in uncontrolled conditions and protected from light. The plot of the P relationship as a function of time (Figure 3) shows that there is no evidence of loss of the determined substance within the entire time interval (0–168 h). At the same time, no additional peaks were observed on the chromatograms, and the statistical analysis confirmed the lack of statistical significance of the slope coefficient (*a*) of the analyzed straight line describing the above-mentioned relationship. Therefore, it can be concluded that the solution of the CS mixture with the internal standard in the mobile phase can be stored under the specified conditions for at least 7 days.

For the confirmation of the equivalent possibility of the use of the HPLC and UV methods to determine CS in solutions with the declared concentration of 2%, CS was determined using both methods, and the results obtained were compared using the *F*-test (Fisher–Snedecor test) and *t*-Student test. The calculated values of both tests, assuming values lower than the critical ones, prove that both methods are not different in terms of the precision of determination, and the calculated CS concentrations are not different in a statistically significant manner (Table 4). However, it should be noted that, in the case of stability analysis of CS solutions, or when their degradation is suspected, the HPLC method should be applied as a selective method for CS determination.

### 2.2. Stress Test

**Figure 4:**
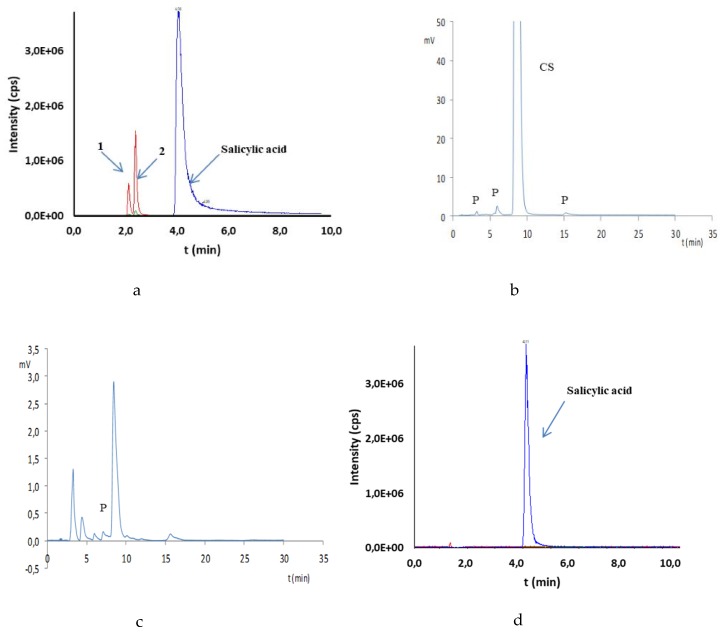
Analysis of CS solution (20 mg/mL) subjected to (**a**) HPLC-MS/MS after oxidation (10% H_2_O_2_, room temp. 24 h), (**b**) HPLC-UV after acidic hydrolysis (1 M HCl, 90 °C, 24 h), (**c**) HPLC-UV after alkaline hydrolysis (1 M NaOH, 90 °C, 24 h), and (**d**) HPLC-MS/MS after alkaline hydrolysis (1 M NaOH, 90 °C, 24 h). 1—2,5-dihydroxybenzoic acid; 2—2,3-dihydroxybenzoic acid; CS—principal peak; P–unidentified products.

The multiple reaction monitoring (MRM) and negative ionization mode were applied for ion transitions monitoring. Due to this, only a priori selected mass transitions were monitored. This selection was performed during method optimization, where the fragmentation processes of compounds of interests were monitored and acquired. In this step of method development, the stock solutions of reference substances were injected and selection of mass transitions were performed. The mass transitions for compound identification are presented in Table 2. Since the 2,3-dihydroxybenzoic acid and 2,5-dihydroxybenzoic acid have the same mass, these compounds were identified as the same mass transitions 152.9 → 108.9 and 152.9 → 80.9. Thus, to obtain the separation of these molecules, the chromatographic separation on the Nucleosil C18 column (4.6 × 150 mm, 5 µm) were applied. Satisfactory separation was obtained. The retention times are presented in Table 2. A chromatogram of both, clearly separated acids is shown, e.g., in Figure 4a. 

In the oxidative environment (H_2_O_2_), the test was performed at room temperature and the loss of CS observed amounted to 7.1% in 10% H_2_O_2_ after 24 h. In the HPLC-UV chromatograms, a phenol peak (6.21 min) consistent with the blank determination test and an unidentified peak with a retention time of 15.24 min were observed (Table 5). Despite the injection of the 1000 ng/mL standard solution, phenol was not visible in HPLC-MS/MS chromatograms. It was recognized that the high temperature present in the ion source can cause its rapid degradation to very small fragments. Thus, this method did not allow confirmation of its presence in the tested mixtures. In contrast, HPLC-MS/MS analysis confirmed the presence of 2,3- and 2,5-dihydroxybenzoic acids as products of oxidation of CS (Table 5, Figure 4a). Under the oxidative conditions, CS was considered as very stable (according to the ICH) (Table 5).

The CS susceptibility test for hydrolytic decomposition was performed at 90 °C. In the neutral environment (water), no CS degradation was observed for 10 days, and as such, CS can be classified as a practically stable substance under these conditions (according to the ICH) (Table 5). In the acidic environment (HCl) at the same temperature, the CS degradation observed occurred at a hydrochloric acid concentration of 1 M (Table 5). With a CS loss of 1.3% after 24 h, the additional unidentified peaks with t_r_ = 3.24, 6.19, and 15.24 min were observed on the HPLC-UV chromatograms (Figure 4b). A peak with a retention time of 6.19 min can be identified as the peak of phenol contaminating the raw material. Such a small loss of CS qualifies it as practically stable in the acidic environment (according to the ICH). On the other hand, in an alkaline medium (NaOH), after 24 h, the CS loss was 36.7% in 1 M NaOH while maintaining stability in 0.1 M NaOH (Table 5). Therefore, CS can be classified as stable in an alkaline environment. The chromatograms of the alkaline CS solutions allowed for observations of additional unidentified peaks at t_r_ = 3.28, 4.40, 6.20, 7.10, and 16.10 min (Figure 4c). The peak characteristic for phenol (6.20 min), in this case, was strengthened compared to the blank test, which may indicate partial decomposition of CS into phenol under these conditions. HPLC-MS/MS analysis did not confirm the presence of 2,3- and 2,5-dihydroxybenzoic acids as well as pyrocatechol and phenol in CS solutions subjected to basic hydrolysis (Figure 4d). No peaks for mass transitions characteristic for these compounds were observed (Table 2).

**Figure 5:**
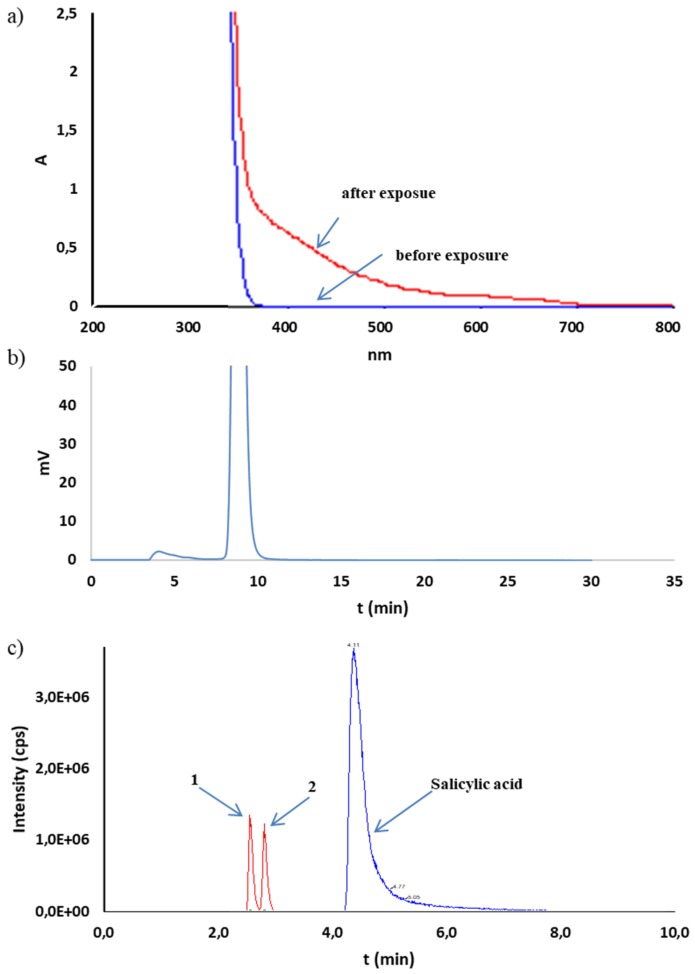
UV (**a**), HPLC-UV (**b**), and HPLC-MS/MS (**c**) analysis of CS solution (20 mg/mL) subjected to photodegradation (1.2 · 10^6^ lux · h). 1—2,5-dihydroxybenzoic acid; 2—2,3-dihydroxybenzoic acid.

**Figure 6:**
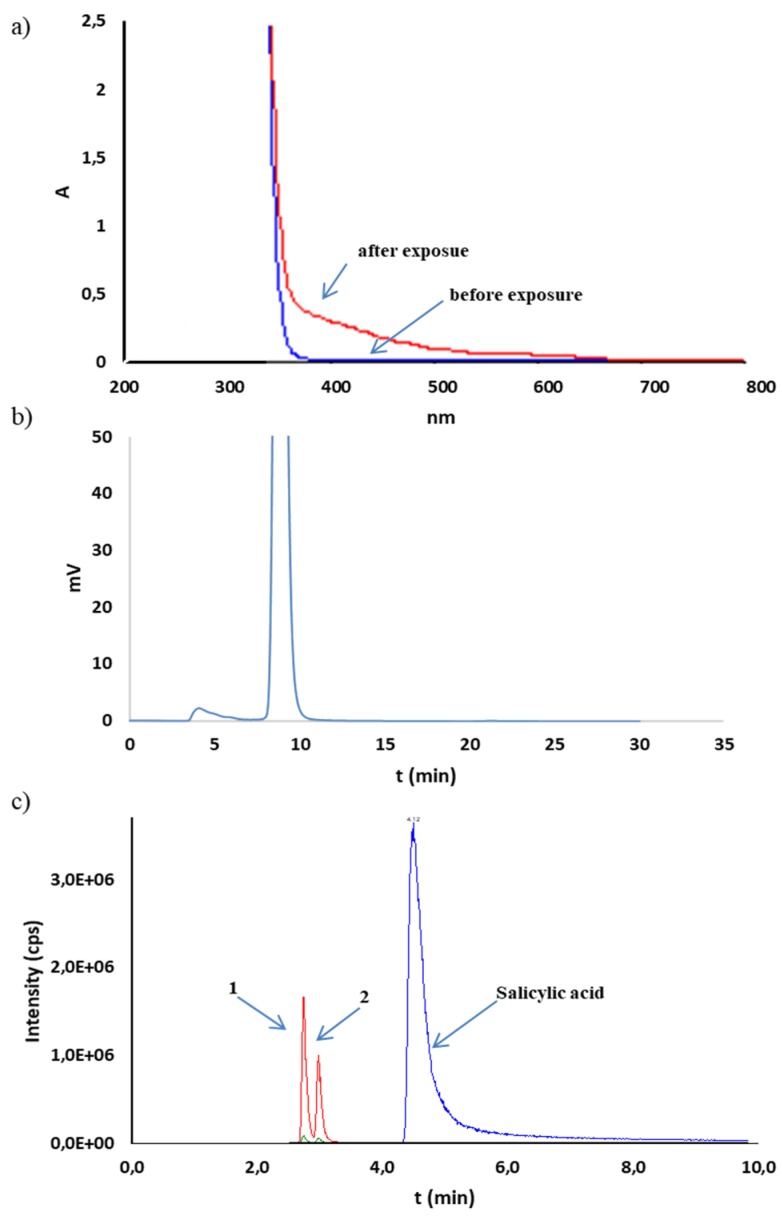
UV (**a**), HPLC-UV (**b**), and HPLC-MS/MS (**c**) analysis of sodium salicylate solution (13 mg/mL) subjected to photodegradation (1.2 · 10^6^ lux · h). 1—2,5-dihydroxybenzoic acid; 2—2,3-dihydroxybenzoic acid.

**Figure 7:**
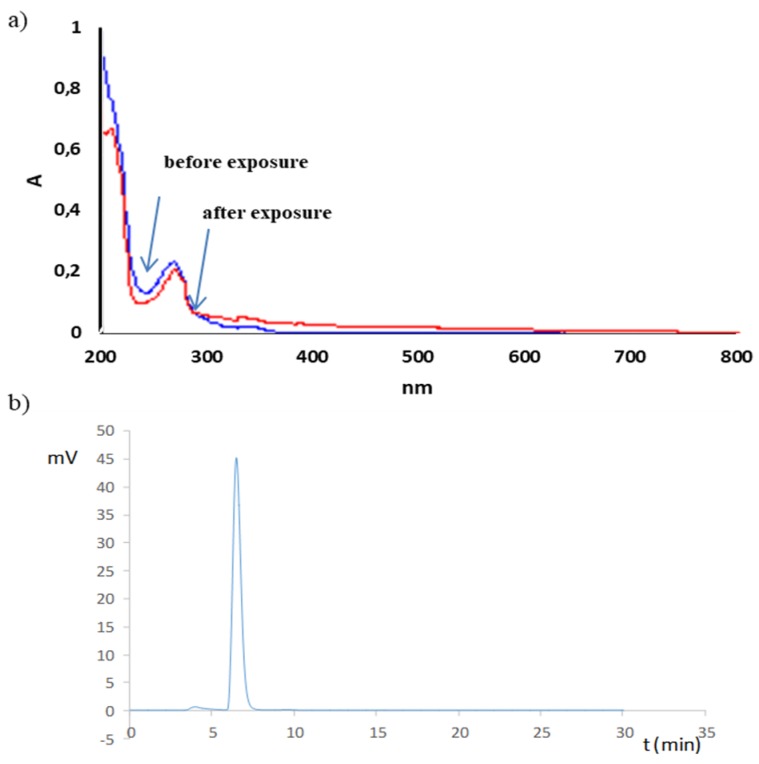
UV (**a**) and HPLC-UV (**b**) analysis of phenol solution (0.5 mg/mL) subjected to photodegradation (1.2 · 10^6^ lux · h).

CS in solution appeared to be susceptible to decomposition by the effect of radiation (photolabile). The samples exposed to radiation at 1.2 and 6.0 · 10^6^ lux·h underwent decomposition at 6.6 and 9.8% (Table 5). Absorbance changes above 350 nm were observed in the UV spectra of CS solutions (20 mg/mL), sodium salicylate (13 mg/mL), and phenol (0.5 mg/mL) irradiated with a dose of 1.2 · 10^6^ lux·h. This confirmed the noticeable color change to brown–yellow in the CS and sodium salicylate solutions (Figure 5a and Figure 6a). In the case of the phenol solution, such a change was not observed (Figure 7a). Simultaneously, outside the main peak, the additional unseparated peaks were observed from 4.8 to 6.3 min on HPLC-UV chromatograms of CS solutions as well as sodium salicylate (Figure 5b and Figure 6b). Chromatograms of the phenol solution showed no additional peaks (Figure 7b). Analysis by HPLC-MS/MS confirmed the presence of mass transition peaks characteristic for 2,3- and 2,5-dihydroxybenzoic acids in CS and sodium salicylate solutions after exposure to light (Figure 5c and Figure 6c). Therefore, the CS solutions should be stored under conditions providing protection from light.

**Figure 8:**
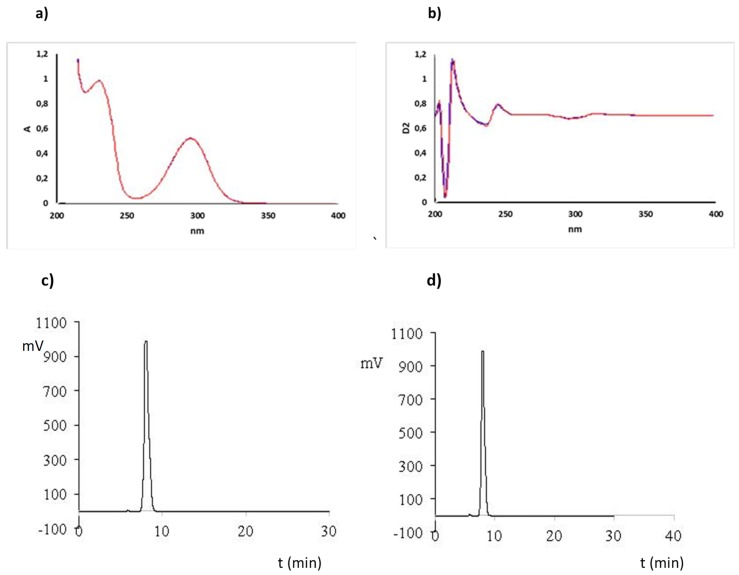
Effect of sterilization of CS solution (2%, g/mL) on UV spectrum (**a**—A, **b**—D2) and HPLC chromatogram before (**c**) and after sterilization (**d**).

The developed UV spectra (A and D2) and the HPLC analysis of solution purity confirmed that CS solutions could be sterilized with saturated steam under pressure at a temperature of 121 ± 2 °C for 20 min and a pressure of 101.4 kPa. The UV spectra showed no changes in the spectrum of the solution before and after sterilization (Figure 8a,b). Similarly, no other additional peaks appeared on the chromatograms, which could indicate the decomposition of CS (Figure 8c,d).

## 3. Materials and Methods

Choline salicylate (CS, 98.4%; Figure 1) was received from manufacturing company ICN Polfa Rzeszów SA, Rzeszów, Poland. Methanol for HPLC and propyl 4-hydroxybenzoate (99%, internal standard, i.s.) were from Avantor Performance Materials Poland S.A. Sodium salicylate was from PharmaCosmetics Fagron (≥ 99.5%), pyrocatechol (≥ 99.5%) and 2,3- (99%) and 2,5-dihydroxybenzoic acid (98%) were from Sigma-Aldrich Sp. z o.o. (Poznań, Poland), and phenol (99%) was from Chempur^®^, Piekary Śląskie, Poland (Figure 1). All other chemicals of the highest purity were commercially available. Demineralized water was used in the tests.

### 3.1. Apparatus and Chromatographic Conditions

#### 3.1.1. HPLC-UV

The high performance liquid chromatography (HPLC) with a system consisting of a Rheodyne 7120, a 20 µL fixed-loop injector, and the Shimadzu liquid chromatograph LC-10AT VP with an SPD-10A VP UV-VIS detector (Shim-Pol, Izabelin, Poland) was used for the determination of CS. As a stationary phase, a Nucleosil 100 C18, 4.6 × 150 mm, 5 μm (MZ Analytical), was used. The mobile phase was a mixture of methanol–water–acetic acid (60:40:1, *v/v/v*). The proposed composition of the mobile phase is a modification of the mobile phase for testing the purity of salicylic acid recommended by Ph. Eur. [24]. The flow rate of the mobile phase was 1.0 mL/min for determination and 0.5 mL/min for purity analysis. The UV detection was carried out at 230 or 270 nm for determination and purity analysis, respectively.

#### 3.1.2. HPLC-MS/MS

LC-MS analyses were performed on a 1260 Infinity system (Agilent Technologies, Santa Clara, CA, USA) with a Nucleosil 100 C18, 4.6 × 150 mm, 5 μm (MZ Analytical) column that was maintained at 20 °C. An isocratic flow at a flow rate of 1 mL/min, with a total run time of 10.0 min, was applied. The mobile phase was a mixture of methanol and water 60:40 (*v/v*) with 1% formic acid as a mobile phase modifier. An injection volume of 5.00 µL was set, with 15 s of needle flushing, with a mobile phase before each injection. The 4000 QTRAP mass spectrometer (Sciex, Framingham, MA, USA) with an electrospray ionization source (ESI) was used for all ion transition monitoring. The multiple reaction monitoring (MRM) and negative ionization mode were applied. A dwell time of 50.0 ms was applied for all transitions monitored. The selection of ion transitions and optimization of the mass spectrometer were performed by infusion of the solutions of the tested compounds dissolved in a mixture of methanol and water, 1:1 (*v/v*), at a 10 µL/min flow rate. Optimization of the ion transition was conducted using an 11 PLUS syringe pump (Harvard Apparatus, Holliston, MA, USA) coupled directly to ESI ion source. The ESI ion source parameters were optimized using flow injection analysis mode. Established ESI ion source parameters were as follows: ion sprays voltage (IS): −4500.0 V; nitrogen curtain gas (CUR): 40.0 psi; temperature (TEM): 600 °C; nitrogen as ion source gas (GS1, GS2): 60.0 psi and 60.0 psi. The selected parameters are shown in Table 2.

Data acquisition and processing were performed under control of Analyst 1.6.3 software (Sciex).

#### 3.1.3. Other Equipment

The UV-Vis spectrophotometer (Lambda 20, Perkin Elmer, Buckinghamshire, UK) with 1 cm matched quartz cells was used for all absorbance measurements in the range from 200 to 400 nm.

The Atlas Suntest CPS + (Atlas, Mount Prospect, IL, USA) and the thermostat ST-1 + (Pol-Eko, Wodzisław Śląski, Poland) were used for the exposure of substances in highly accelerated and stress testing. The irradiation chamber was equipped with a temperature control system (35–100 °C), 1500 W air-cooled xenon lamps, and direct setting and control of irradiance in a wavelength range of 300–800 nm. A photostability study of solutions of the tested substances was performed in quartz cuvettes with a stopper, 10 mm, 3 mL (Merck, Darmstadt, Germany).

Stress tests were performed in a Thermal Testing Chamber KBC-100 (Wamed, Warsaw, Poland) with an accuracy of heat set temperature of ±1.0 °C.

The solutions were sterilized using a small water steam sterilizer (Extacta, M.O. Com S.R.L., Montecchio Emilia, Italy).

### 3.2. Validation of the HPLC Method

#### 3.2.1. Selectivity

A solution of CS (80 μg/mL) with its impurities—4-hydroxybenzoic acid (50 μg/mL) and phenol (50 μg/mL)—and with propyl 4-hydroxybenzoate as an internal standard (50 μg/mL) in the mobile phase was applied to a chromatography column. CS chromatograms in mixture with 4-hydroxybenzoic acid and phenol were developed at a mobile phase flow rate of 1.0 mL/min and with detection at 270 nm (Figure 2a). However, for determination of CS in mixtures with the internal standard, a 1.0 mL/min mobile phase flow rate and 230 nm detection were used (Figure 2b). Chromatograms of potential degradation products—2,3-, 2,5-dihydroxybenzoic acid and pyrocatechol—at a concentration of approximately 50 μg/mL were plotted under conditions of CS purity analysis. The retention times of all reference substances are given in Table 1.

#### 3.2.2. Stability of the Analyte

A solution of CS mixture (80 μg/mL) and internal standard (100 μg/mL) was prepared in a mobile phase and stored under uncontrolled conditions at room temperature protected from light. A solution of the analyte was applied to column chromatography (a mobile phase flow of 1.0 mL/min, and detection of 230 nm) after 24, 48, 72, 120, 144 and 168 h of storage, and the dependence of the ratio of the CS peak area to that of the internal standard (P = P_CS_/P_i.s._) was analyzed as a function of time (Figure 3).

#### 3.2.3. Calibration Curve

Standard curves for 3 series of 9 solutions of CS in the mobile phase at concentrations from 3.94 to 119.1 μg/mL containing a constant concentration of internal standard (propyl 4-hydroxybenzoate, 100 μg/mL) were prepared. Each solution was applied to the column 3 times, and the dependence of the linear P dependence as a function of the concentration of CS in the solution was analyzed. Statistical parameters of simple y = *a*x + *b* and y = *a*x (Table 3) were calculated. The LOD (limit of detection) and LOQ (limit of quantitation) parameters values were calculated from the following formulas:

(1) $$LOD\left(LOQ\right)=\frac{\kappa \cdot SD_{b}}{a}$$

where *κ* is 3.3 for LOD and 10 for LOQ, *SD_b_* is the standard deviation of the intercept, and *a* is the slope y = *a*x + *b*.

#### 3.2.4. Linearity

The linearity of the developed method for the determination of 2% CS solutions was evaluated. Five CS solutions were prepared in a concentration range of 50–150% of the declared value: 1.00, 1.51, 2.01, 2.50, and 3.01% (g/mL). An amount of 2.5 mL of i.s. (1 mg/mL) in methanol was added to 0.1 mL of each CS solution and supplemented with a mobile phase, increasing the total amount up to 25.0 mL. The CS content was determined by HPLC (mobile phase flow: 1.0 mL/min; detection: 230 nm). Each solution was applied to the column 3 times, and the dependence of the linear P dependence as a function of the concentration of CS in the solution was analyzed (Table 3).

#### 2.2.5. Precision, Accuracy, and Reproducibility

CS solutions were prepared in water at concentrations of 1.00, 2.01, and 3.01%, and CS determinations were made by the HPLC method using an internal standard (mobile phase flow: 1.0 mL/min, detection: 230 nm). An amount of 2.5 mL of i.s. (1 mg/mL) in methanol was added to 0.1 mL of each CS solution, and the total amount was increased up to 25.0 mL with a mobile phase. The study was performed over two days (inter-day and intra-day), and the test was repeated six times for each series of solutions (Table 4).

### 3.3. Validation of the UV Method

#### 3.3.1. Calibration Curve

Standard curves for 3 series of 9 solutions of CS in water at concentrations of 2.52–80.56 μg/mL were prepared. UV spectra were plotted 3 times in the range 220–400 nm, and the linear dependence of absorbance at the analytical wavelength of 276 nm was analyzed as a function of the concentration of CS in solution. Statistical parameters of simple y = *a*x + *b* and y = *a*x (Table 3) were calculated. LOD (limit of detection) and LOQ (limit of quantitation) values were calculated using Equation (1).

#### 3.3.2. Linearity

The linearity of the developed method for the determination of 2% CS solutions was evaluated. Five CS solutions were prepared in the concentration range of 50–150% of the declared value: 1.00, 1.51, 2.01, 2.50, and 3.01%. A total of 0.2 mL of each CS solution was supplemented with water for a total of 100.0 mL. The absorbance of each solution (A_276_) was measured 3 times, and the linear dependence of A_276_ as a function of the concentration of CS in solution was analyzed (Table 3).

#### 3.3.3. Precision, Accuracy, and Reproducibility

CS solutions were prepared in water at concentrations of 1.00, 2.01, and 3.01%, and CS determinations were made by the UV method. A total of 0.2 mL of each CS solution was supplemented with water for a total of 100.0 mL. The study was performed over two days (inter-day and intra-day), and the test was repeated six times for each series of solutions (Table 4).

### 3.4. Comparison of HPLC and UV Methods

A solution of CS (2%) was prepared, and the content of CS was determined using HPLC and UV methods, repeating the assay 6 times. The CS content in the solution was calculated using the parameters of the calibration curve (Table 4).

### 3.5. Stress Test

#### 3.5.1. Strong Acid, Strong Base Degradation, and Neutral Hydrolysis

Solutions of CS (20 mg/mL) in water and 0.1 M hydrochloric acid as well as CS (200 μg/mL) in a 1.0 M hydrochloric acid or sodium hydroxide solution (0.1 and 1.0 M) were prepared and transferred to sealed vials (10 mL). The tubes were stored at a temperature indicated in Table 5. After the appropriate time, the samples were cooled to room temperature and diluted in a 1:1 ratio of water or with NaOH/HCl (0.1 and 1.0 M) to neutralize it. Half of the samples were then applied to the chromatography column to register all products of decomposition (purity analysis). For this purpose, the solutions were diluted with the mobile phase to obtain a concentration of CS, c.a. 5 mg/mL. Other samples were added to the solution of the internal standard and diluted with the mobile phase to obtain a CS concentration of c.a. 80 µg/mL and a 100 µg/mL internal standard. Solutions prepared in this way were applied to the chromatography column for quantitative analysis of CS. Comparative chromatograms of mixtures of CS solutions (80 µg/mL) and the internal standard (100 µg/mL) in the mobile phase were plotted. Each sample was analyzed thrice, and the content of the tested substance in the solution was calculated as a percentage of the declared value (Table 5). CS solutions subjected to acid and base hydrolysis, after their neutralization, were also analyzed with HPLC-MS/MS.

#### 3.5.2. Oxidative Degradation

Solutions of CS (20 mg/mL) in hydrogen peroxide (3 and 10%) were prepared and placed in sealed tubes (10 mL). The tubes were protected from light and stored at room temperature. After an appropriate time, the purity of the solutions was evaluated with the HPLC methods, and the CS content was determined with the HPLC-UV method according to the previously performed procedure (Table 5).

#### 3.5.3. Photostability

An amount of 20.0 mL of aqueous solutions of the CS (20 mg/mL) was prepared, placed in quartz cuvettes (l = 1 cm) and stored 22 h or 109 h in the Atlas Suntest CPS+ (250 W·m^−2^; 1.2 · 10^6^ lux·h or 6.0 · 10^6^ lux·h, respectively). For each series of tested substances, two samples were prepared: protected from light (cuvettes were protected with aluminum foil) and unprotected. At time t = 0 and after exposure, an HPLC analysis was carried out: purity analysis and CS determination (Table 5). Simultaneously, 0.2 mL of each CS solution were supplemented with water to total 100.0 mL, and a UV spectrum was plotted. An aqueous solution of sodium salicylate (13 mg/mL) and phenol (0.5 mg/mL) was also subjected to a photodegradation process. UV spectra and HPLC-UV chromatograms were plotted as in the case of the CS solutions. After exposure, HPLC-MS/MS analyses of CS and sodium salicylate solutions were also conducted.

#### 3.5.4. CS Stability Under Sterilization Conditions

The CS solution (2%, g/mL) was sterilized with saturated steam at 121 ± 2 °C for 20 min under 101.4 kPa, and after an appropriate dilution, UV spectra (A = absorbance; D_2_ = second derivative of absorbance) and purity by the HPLC-UV method were determined.

## 4. Conclusions

Within the range of the concentrations being analyzed, both methods (HPLC-UV and UV) were suitable to determine CS accurately and in a reproducible manner. Due to the lack of selectivity, the UV method can be used to determine CS in preformulative cases, e.g., the release or permeability of CS, as a fast, inexpensive, and sensitive method. By contrast, the HPLC-UV method, which is selective, allows CS to be determined in the presence of its potential decomposition products, impurities, and the internal standard (propyl 4-hydroxybenzoate). According to the ICH guidelines, the tested compound was classified as follows: practically stable in a neutral and acidic environment, stable in an alkaline environment, very stable in an oxidative environment, and photolabile in solution. The HPLC-MS/MS method confirmed that 2,3- and 2,5-dihydroxycarboxylic acids are degradation products of CS after oxidation and after irradiation. The CS solutions can be sterilized at an elevated temperature (121 ± 2 °C) for 20 min, using saturated steam at a pressure of 101.4 kPa, but they should be stored in packaging protected from light.

## Figures and Tables

**Table 1:** Comparison of retention times of standard solutions of potential impurities and degradation products observed under conditions of CS purity analysis by HPLC-UV.

Compound	Retention Time, min	Compound	Retention Time, min
4-Hydroksybenzoic acid	4.51	2,3-Dihydroxybenzoic acid	5.48
Pyrocatechol	5.02	Phenol	6.24
2,5-Dihydroxybenzoic acid	5.08	Choline salicylate	8.85

**Table 2:** Parameters optimized for the MS/MS analyses.

Compound	Molecular Weight, Da	Precursor Ion, m/z	MRM Transition, m/z	Retention Time, min	DP, V	EP, V	CE, V	CXP, V
2,3-Dihydroxybenzoic acid	154.1	152.9	152.9→108.9	2.47	−30	−8	−22	−15
			152.9→80.9				−30	−15
2,5-Dihydroxybenzoic acid			152.9→108.9	2.21			−22	−15
			152.9→80.9				−30	−15
Salicylic acid	138.1	136.9	136.9→92.9	4.13	−30	−8	−25	−15
			136.9→65.0				−45	−15
Pyrocatechol	110.0	108.9	108.9→90.9	2.15	−70	−8	−27	−15
			108.9→80.9				−24	−15
Phenol	94.1	92.9	92.9→74.9	-	−80	−8	−30	−12
			92.9→65.0				−28	−10

DP—declustering potential; EP—entrance potential; CE—collision energy; CXP—collision cell exit potential.

**Table 3:** The quantitative parameters and statistical data for determination of CS by the proposed HPLC and UV method—validation parameters: calibration curves and linearity.

**Calibration Curves**						
**Method**	**Range, µg/mL**	**n**	**Statistical Parameters**		**LOD, µg/mL**	**LOQ, µg/mL**
			**y = *a*x + *b***	**y = *a*x**		
HPLC	3.94–119.10	9	*a* = (1.10 ± 0.01) 10^−2^	*a* = (1.10 ± 0.01) 10^−2^	1.16	3.51
			*b* = (5.75 ± 9.13) 10^−3^	*r* = 0.9999		
			*r* = 0.9999			
			*t_b_* = 1.486 < t_0.05_ (7) = 2.365			
UV	2.52–80.56	14	*a* = (1.34 ± 0.01) 10^−2^	a = (1.34 ± 0.01) 10^−2^	0.43	1.30
			*b* = (−3.74 ± 3.82) 10^−3^	*r* = 0.9999		
			*r* = 0.9999			
			*t_b_* = −2.130 < *t*_0.05_ (12) = 2.179			
**Linearity**						
**Method**	**Range, %**	**n**	**Statistical Parameters**			
			**y = *a*x + *b***	**y = *a*x**		
HPLC	1.00–3.01	5	*a* = 0.436 ± 0.032	*a* = 0.444 ± 0.024		
			*b* = (1.82 ± 6.91) 10^2^	r = 0.9992		
			*r* = 0.9992			
			*t_b_* = 0.837 < t_0.05_ (3) = 3.182			
UV	1.00–3.01	5	*a* = 0.267 ± 0.010	*a* = (1.34 ± 0.01) 10^−2^		
			*b* = (6.16 ± 22.41) 10^−3^	*r* = 0.9998		
			*r* = 0.9998			
			*t_b_* = 0.874 < *t*_0.05_ (3) = 3.182			

**Table 4:** The quantitative parameters and statistical data for determination of CS by the proposed HPLC and UV method—validation parameters: precision, accuracy, and comparative analysis.

Precision and Accuracy							
Method	Declared Content, %	Amount Found, % (Recovery, %)					Repeatability
		Inter-Day		RSD, %	Intra-Day	RSD, %	RSD_r_, %
HPLC	1.00	1.01 ± 0.01 (101.0 ± 1.4)		1.33	1.01 ± 0.02 (101.0 ± 2.0)	1.86	0.40
	2.01	2.01 ± 0.03 (100.2 ± 1.3)		1.26	2.00 ± 0.03 (99.6 ± 1.6)	1.56	0.53
	3.01	3.02 ± 0.02 (100.4 ± 0.9)		0.82	3.05 ± 0.02 (101.5 ± 0.8)	0.73	0.25
UV	1.00	1.00 ± 0.01 (101.0 ± 1.4)		1.26	0.99 ± 0.01 (101.0 ± 1.4)	1.20	0.31
	2.01	2.04 ± 0.01 (100.2 ± 1.3)		0.43	2.03 ± 0.01 (100.2 ± 1.3)	0.55	0.19
	3.01	3.02 ± 0.02 (100.4 ± 0.9)		0.67	2.99 ± 0.01 (100.4 ± 0.9)	0.32	0.11
**Comparative Analysis of HPLC and UV Method**							
**Method**	**x ± Δx, %**	**SD, %**	**SDx, %**	**RSD, %**	***F*-Snedecor Test**	***t*-Student Test**	
HPLC	2.01 ± 0.03	2.54 · 10^−2^	1.04 · 10^−2^	1.26	*F* = 0.52	*t* = 0.371	
UV	2.02 ± 0.02	1.82 · 10^−2^	7.45 · 10^−3^	0.90	*F*_0.05_(5;5) = 5.05	*t*_0.05_(10) = 2.228	

**Table 5:** The content of the CS in solutions after hydrolysis and oxidation, calculated in relation to the declared value and the retention times of the observed degradation products designated by HPLC methods.

Medium	x ± Δx, %	SD, %	SDx, %	RSD, %	HPLC t_r_, min (Compound or Part in %)
**Hydrolysis in a Neutral Environment**					
H_2_O 90 °C, 36 h	95.8 ± 5.7	2.29	1.32	2.39	HPLC-UV: no additional peaks
H_2_O 90 °C, 2 days	102.7 ± 10.0	4.01	2.32	3.91	HPLC-UV: no additional peaks
H_2_O 90 °C, 4 days	99.3 ± 18.7	7.51	4.34	7.57	HPLC-UV: no additional peaks
H_2_O 90 °C, 10 days	103.7 ± 6.7	2.70	1.56	2.60	HPLC-UV: no additional peaks
**Hydrolysis in an Acidic Environment**					
HCl 0.1 M 90 °C, 24 h	99.5 ± 3.1	1.27	0.732	1.27	HPLC-UV: no additional peaks
HCl 1 M 90 °C, 24 h	98.7 ± 5.4	2.20	1.27	2.22	HPLC-UV: 3.24 (0.18), 6.19 (0.08), 15.24 (0.08)
**Hydrolysis in an Alkaline Environment**					
NaOH 0.1 M 90 °C, 24 h	102.0 ± 1.9	0.779	0.450	0.76	HPLC-UV: no additional peaks
NaOH 1 M 90 °C, 24 h	63.3 ± 2.0	0.807	0.466	1.28	HPLC-UV: 3.28 (20.5), 4.40 (7.3), 6.20 (1.4) 7.10 (1.4), 16.10 (1.4) HPLC-MS/MS *
**Decomposition in an Oxidizing Environment**					
3% H_2_O_2_ room temp., 6 h	97.2 ± 1.2	0.490	0.283	0.50	HPLC-UV: no additional peaks
3% H_2_O_2_ room temp., 24 h	102.3 ± 3.0	1.19	0.685	1.17	HPLC-UV: no additional peaks
10% H_2_O_2_ room temp., 24 h	92.9 ± 0.3	0.124	0.0715	0.13	HPLC-UV: 6.21 (0.2), 15.24 (0.8) HPLC-MS/MS: 2.21 (2,5-dihydroxybenzoic acid) 2.47 (2,3-dihydroxybenzoic acid)
**Photostability in Solution (1.2 · 10^6^ lux·h)**					
protected	100.0 ± 9.3	3.73	2.15	3.73	HPLC-UV: no additional peaks
not protected	93.4 ± 1.9	0.769	0.444	0.82	HPLC-MS/MS: 2.21 (2,5-dihydroxybenzoic acid) 2.47 (2,3-dihydroxybenzoic acid)
**Photostability in Solution (6.0 · 10^6^ lux·h)**					
protected	99.6 ± 2.5	1.02	0.590	1.02	HPLC-UV: no additional peaks
not protected	90.2 ± 0.3	0.137	0.0791	0.15	HPLC-MS/MS: 2.21 (2,5-dihydroxybenzoic acid) 2.47 (2,3-dihydroxybenzoic acid)

* the presence of 2,5- and 2,3-dihydroxybenzoic acids and pyrocatechol has not been confirmed.
